# Fit to WHO weight standard of European infants over time

**DOI:** 10.1136/archdischild-2015-309594

**Published:** 2016-02-16

**Authors:** Daniel Levin, Louise Marryat, Tim J Cole, John McColl, Ulla Harjunmaa, Per Ashorn, Charlotte Wright

**Affiliations:** 1 School of Mathematics and Statistics, University of Glasgow, Glasgow, UK; 2 Population, Policy and Practice Programme, UCL Institute of Child Health, London, UK; 3 Department for International Health, University of Tampere School of Medicine, Tampere, Finland; 4 Department of Paediatrics, Tampere University Hospital, Tampere, Finland; 5 Department of Child Health, University of Glasgow, Queen Elizabeth University Hospital, Glasgow, UK

**Keywords:** Epidemiology, General Paediatrics, Growth, Obesity, Nutrition

## Abstract

**Objectives:**

The 2006 WHO growth charts were created to provide an international standard for optimal growth, based on healthy, breastfed populations, but it has been suggested that Northern European children fit them poorly. This study uses infant weight data spanning 50 years to determine how well-nourished preschool children from different eras fit the WHO standard, and discuss the implications of deviations.

**Design:**

Four longitudinal datasets from the UK and one from Finland were used comprising over 8000 children born between1959 and 2003. Weights from birth to 2 years were converted to age–sex-adjusted Z scores using the WHO standard and summarised using Generalized Additive Models for Location, Scale and Shape.

**Results:**

Weights showed a variable fit to the WHO standard. Mean weights for all cohorts were above the WHO median at birth, but dipped by up to 0.5 SD to a nadir at 8 weeks before rising again. Birth weights increased in successive cohorts and the initial dip became slightly shallower. By age 1 year, cohorts were up to 0.75 SD above the WHO median, but there was no consistent pattern by era.

**Conclusions:**

The WHO standard shows an acceptable, but variable fit for Northern European infants. While birth weights increased over time, there was, unexpectedly, no consistent variation by cohort beyond this initial period. Discrepancies in weight from the standard may reflect differences in measurement protocol and trends in infant feeding practice.

**What is already known on this topic**

- Infants in high-income countries tend to fit the WHO 2006 growth standard well for length.
- Infants do, however, become heavier than the standard after 6 months.

**What this study adds**

- Northern European infants demonstrate a largely adequate fit to the WHO weight standard after the first 2 weeks.
- Infants born recently fit the WHO standard at birth and in the early weeks better than earlier cohorts.
- All the cohorts were heavier than the WHO standards by age 12 months, with no trend over time, suggesting that this cannot be explained by increasing rates of population obesity.

## Introduction

Growth charts are widely used in child health to identify undernutrition and overnutrition.^1^ Many countries produce their own charts describing how local children grow. However, these do not necessarily characterise healthy growth; in some countries average weight will be low due to undernutrition, while in high-income countries higher average weights reflect obesity. In the past, charts were based predominantly on bottle-fed infants and did not adequately reflect the growth of breastfed infants who should be the physiological norm.^2^ Thus, the WHO developed new growth charts based on healthy breastfed children living in optimal circumstances in six world regions.^3^ The WHO showed that linear growth differed little between the six datasets^4^ and thus argued that they could be used to define how all children aged 0–5 years *should* grow, whatever their ethnic origin. However, weights by country for the WHO dataset have never been published. Since the publication of the standard, studies in unselected, healthy, non-deprived populations such as the UK,^5^ USA,^6^ Canada,^7^ Norway, Belgium,^8^ Italy, Argentina^9^ and Denmark^10^ have generally found a close fit for length, but a tendency for children to become heavier than the WHO standard after the first 6 months. Some authors have argued that these discrepancies represent a fundamental difference that renders the standard unsuitable for high-income countries.^8^ Others have suggested that variations in fit are to be expected if the WHO charts represent optimal rather than average growth, as few children will be breastfed to age 1 year^11^ and that the higher weights reflect rising rates of obesity at all ages.^5^

If the mismatch *is* due to obesity, it ought to be less evident in historic datasets from eras when obesity was less common. Conversely, if it reflects the nature, prevalence and duration of formula feeding, the fit should be better in more recent cohorts as breastfeeding has increased.

### Aims

Our aims are to explore a portfolio of weight datasets from the past 50 years (i) to determine how well real populations of well-nourished preschool children from different eras fit the 2006 WHO growth standard at different ages during infancy; and (ii) to explore how trends differ by era of the cohort and associated infant feeding patterns.

## Methods

### Datasets

Data came from existing longitudinal growth studies, retrieved mainly from routine records. They had already been cleaned, checked and analysed for other purposes, with four studies already published. Details of the five studies are as follows:

#### Widdowson study (1959)

In a study set up by Dr Elsie Widdowson, routine weights of 1094 babies born in 1959–1965 were obtained contemporaneously from the records of 10 Cambridge Child Welfare Clinics. Weights were measured by clinical staff approximately monthly in the first year of life, with a maximum of 13 weights per child. Of the possible 14 222 measurements all but 864 (6%) were collected. Although the data were cleaned and analysed at the time of collection the results were never published.

#### Cambridge Infant Growth study (1984)

Cambridge Infant Growth Study (CIGS) was a research cohort of 255 babies recruited in 1984–1987. Infants were sampled in four cohorts from lists of Cambridge city mothers booked to deliver in particular months, with some filtering by midwives. Measurements were taken mainly by one highly trained auxologist every 4 weeks from 4 to 52 weeks, then at 15, 18, 24, 30, 36 and 48 months. Weight, length, head and arm circumferences, triceps and subscapular skinfolds were measured at each visit: 223 (87%) had all 15 measurements from birth to 2 years.^12–14^

#### Newcastle Growth and Development study (1987)

This dataset comprises the routine weights of a birth cohort of 3418 children born at term in Newcastle upon Tyne between June 1987 and May 1988. Up to 11 weights measured by clinical staff in infancy were retrieved from baby clinic records, and 3060 of the babies had at least two weights.^15^ ^16^

#### Gateshead Millennium study (1999)

Gateshead Millennium Study (GMS) is a birth cohort of 1029 babies (923 term) born in Gateshead in 1999–2000, representing 81% of eligible births during the recruitment period. Routine weights were retrieved from baby clinic records. There was a mean of 13 weights per child in the first year. Research nurses measured 830 infants at 13 months.^17–19^

#### Tampere study, Finland (2003)

This dataset comprises the routine heights and weights of 2809 children aged 0–4 years born between October 2003 and September 2004 who attended child health clinics in Tampere. Children were weighed by clinical staff on electronic scales. Up to 16 scheduled events were recorded per child at birth, 1–2 weeks, 6–8 weeks, 2, 3, 4, 5, 6, 8, 10, 12, 18, 24, 36 and 48 months. There was a mean of 12 measurements per child.^20^

### Analysis

The datasets were cleaned and weights converted into Z scores relative to the WHO growth standard. Data beyond 2 years were excluded, when numbers were low and bias was likely. The measurements at birth and 6–8 weeks, 3, 6, 9, 12 18 and 24 months were summarised by age, sex and cohort (table 1). The mean Z score, SD, skewness and kurtosis were modelled as functions of age for each dataset by sex, using Generalized Additive Models for Location, Scale and Shape (GAMLSS) as implemented in the GAMLSS package in R V.3.1.1. Multiple GAMLSS models were fitted using different hyperparameters and distribution families. In the final models, based on the Box-Cox power exponential family (BCPE), the mean Z score was allowed to vary with age in each of the datasets, whereas the SD, skewness and kurtosis were constrained to be constant as exploratory analyses showed this made little difference to the model fit as determined using the Bayesian Information Criterion. The skewness adjustment ensured that the mean and median were effectively the same. The BCPE requires values to be positive, so all Z scores had 10 added to them prior to analysis, and 10 was then subtracted from the mean curves. The models for each cohort were plotted as mean Z score versus age in boys and girls, along with the constant SD, skewness and kurtosis, to compare how well the cohorts fitted the WHO 2006 standard. A mean Z score of 0, SD 1, skewness 1 and kurtosis 2 indicates a perfect fit, whereas a mean Z score above 0 is heavier than the WHO standard, and below is lighter.

**Table 1:** Number of observations at target ages by dataset

			Number of observations				
Target age (weeks)	Age range	Sex	Widdowson (1959)	CIGS (1984)	GDS (1987)	GMS (1999)	Tampere (2003)
0	Birth	M	573	142	1589	484	929
		F	519	122	1582	477	889
7	5–9 weeks	M	452	135	1201	388	1202
		F	410	116	1217	379	1145
13	2–4 months	M	573	137	1357	392	1220
		F	520	118	1365	389	1189
26	5–7 months	M	570	135	1186	240	1192
		F	516	117	1212	247	1174
39	8–10 months	M	569	134	898	222	993
		F	515	116	893	233	962
52	11–14 months	M	501	131	1068	308	1161
		F	476	116	1109	300	1134
78	15–21 months	M	–	125	642	61	1132
		F	–	116	670	66	1098
104	22–30 months	M	–	124	122	–	1076
		F	–	115	115	–	1068

CIGS, Cambridge Infant Growth Study; GDS, Growth and Development Study; GMS, Gateshead Millennium Study.

There is no standard definition of what constitutes a good or poor fit to a growth reference. In *this* study fit was defined in terms of mean weight Z score, measured in fractions of a centile channel width relative to the WHO median, where one channel width=0.67 SDs.^21^ An excellent fit was defined as a difference of no more than ¼ of a channel width (0.17 SD) and a poor fit as greater than a channel width (0.67 SD).

## Results

Mean birthweight Z scores in the five datasets were all positive and close to zero (table 2). They were progressively higher in later years, particularly in the boys, rising from 0.03 in 1959 to 0.37 in 2003. For all cohorts the fit was adequate in early infancy, mostly staying within half a channel width of the median, but by 1 year boys and girls in Widdowson and boys in Tampere fitted poorly, more than a channel width above the median.

**Table 2:** Mean (SD) weight Z scores at target ages by dataset

		Mean (SD) weight Z score of children by age, sex and study cohort, Z-score units				
Target age (weeks)	Sex	Widdowson (1959)	CIGS (1984)	GDS (1987)	GMS (1999)	Tampere (2003)
0	M	**0.03** **(1.11)**	**0.06 (0.97)**	**0.12 (1.01)**	0.18 (1.05)	0.37 (1.08)
	F	**0.10 (1.01)**	0.30 (0.89)	**0.08 (1.07)**	**0.17 (1.1)**	0.40 (1.1)
7	M	−0.40 (1.02)	−0.28 (0.99)	−0.25 (0.95)	**−0.16 (0.96)**	**0.14 (1.05)**
	F	−0.30 (0.83)	−0.19 (0.83)	−0.30 (0.91)	**−0.17 (0.9)**	**0.12 (0.95)**
13	M	−0.33 (1.07)	−0.34 (0.97)	−0.21 (0.98)	**−0.16 (0.97)**	**0.17 (1.03)**
	F	**−0.17 (0.83)**	−0.22 (0.86)	−0.29 (0.9)	−0.18 (0.9)	**0.12 (0.91)**
26	M	0.28 (0.99)	**−0.09 (0.98)**	**0.15 (1.01)**	0.30 (0.99)	0.46 (1.07)
	F	0.42 (0.82)	**0.05 (0.87)**	**0.05 (0.91)**	0.21 (0.93)	0.38 (0.93)
39	M	0.62 (1)	**0.05 (0.97)**	0.38 (1.05)	0.56 (1.01)	0.56 (1.09)
	F	*0.67 (0.8)*	0.21 (0.86)	0.23 (0.93)	0.43 (0.92)	0.48 (0.96)
52	M	*0.69 (0.97)*	**0.16 (0.97)**	0.53 (1.01)	0.55 (1.06)	*0.60 (1.01)*
	F	*0.76 (0.81)*	0.26 (0.83)	0.38 (0.89)	0.51 (0.91)	0.52 (0.91)
78	M	–	0.20 (0.94)	0.46 (1)	0.44 (0.97)	*0.62 (0.96)*
	F	–	0.23 (0.76)	0.27 (0.95)	0.41 (1.01)	0.56 (0.89)
104	M	–	**0.04 (0.96)**	0.38 (1.12)	–	0.52 (0.96)
	F	–	0.18 (0.71)	**0.12 (1.12)**	–	0.53 (0.91)

Fit: **excellent (≤0.17 SD)**, poor *(>0.67 SD)*.

CIGS, Cambridge Infant Growth Study; GDS, Growth and Development Study; GMS, Gateshead Millennium Study.

**Figure 1:**
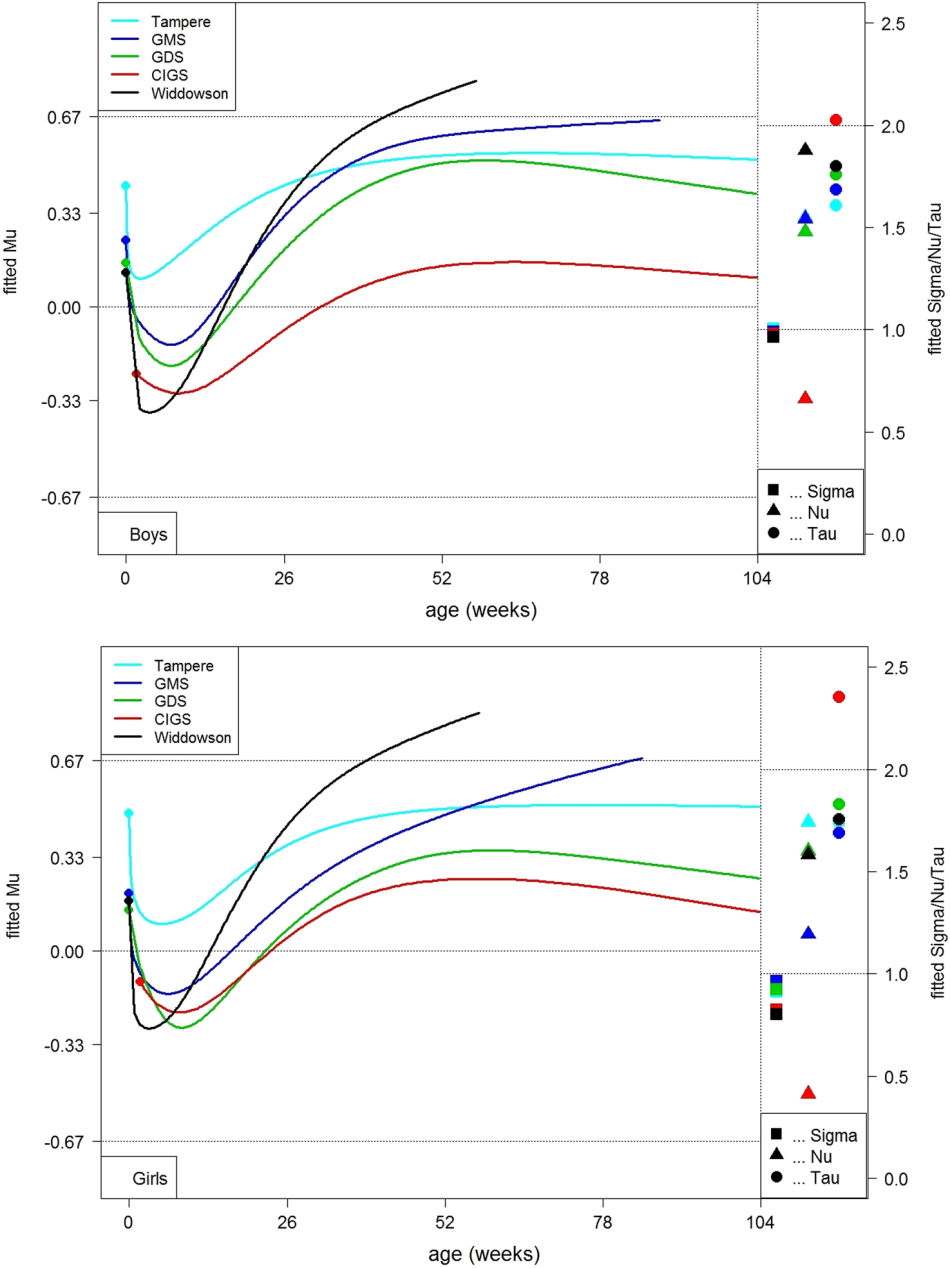
Mean weight Z scores by age in five Northern European cohorts (top boys; bottom girls). CIGS, Cambridge Infant Growth Study; GDS, Growth and Development Study; GMS, Gateshead Millennium Study.

The smoothed curves of mean Z score versus age from the GAMLSS models were plotted by cohort on separate charts by sex, along with the constant SD (sigma), skewness (nu) and kurtosis (tau) (figure 1). The curves provide a visual assessment of the fit to the standard by cohort. All datasets showed consistent differences relative to the standard. The mean Z scores all tended to fall in the early weeks, with the four UK datasets ending up below zero. This steep fall was followed by a slightly less steep rise, creating a ‘trough’ in each curve at around 8 weeks. Boys and girls followed broadly the same pattern.

The depth of the trough, like birthweight, became progressively shallower over time. Mean Z score fell by between 0.2 and 0.5 SDs from birth to 7 weeks, the fall tending to be greater in the earlier cohorts (table 3). The earliest dataset (Widdowson) also showed the steepest rise in Z score after the trough, so that by 52 weeks it was the heaviest, around a channel width above the median. In contrast, the second earliest dataset (CIGS) rose least and remained close to the median. Mean Z scores in the remaining datasets rose by around 0.5 SD and tended to level out after 52 weeks. It should be noted that the Widdowson and GMS datasets had only limited data in later infancy, and thus the trajectory curves are incomplete. Patterns for the girls and boys were similar (figure 1). The fitted constant SDs showed a good fit relative to the WHO standard, being close to 1 throughout. Skewness was less consistent, with negative skewness (nu <1) in CIGS and positive skewness (nu >1) in the other datasets. Kurtosis was near 2 as expected.

**Table 3:** Change in mean (SD) weight Z scores between target ages by dataset

		Change in SDS				
Target age (weeks)	Sex	Widdowson (1959)	CIGS (1984)	GDS (1987)	GMS (1999)	Tampere (2003)
0–7	M	−0.43	−0.34	−0.37	−0.34	−0.23
	F	−0.40	−0.49	−0.38	−0.34	−0.28
7–52	M	1.09	0.44	0.78	0.71	0.46
	F	1.06	0.45	0.68	0.68	0.40

CIGS, Cambridge Infant Growth Study; GDS, Growth and Development Study; GMS, Gateshead Millennium Study.

## Discussion

The strengths of this study are that the five datasets represent weight gain before and during the obesity epidemic. The datasets are of high quality and all but CIGS are representative of their populations, with high recruitment rates. The GAMLSS modelling approach makes maximum use of the available data and controls for potential bias. A weakness is that the most contemporary study is from Finland, not the UK, where growth patterns may differ systematically.

Overall, most cohorts were within a channel width of the WHO median and many showed an excellent fit in the early weeks, though less so beyond this point. It seems unlikely that these later differences could be simply genetic, since two of the six WHO datasets were of predominantly Northern European origin and they showed no consistent differences in stature.^22^ The lack of variation by era argues against obesity being the explanation.

The increase in birthweight over time is consistent with research in other high-income countries.^3^ ^22^ ^23^ This is generally thought to reflect less maternal cigarette smoking and more maternal obesity and gestational diabetes.^24^ It has been suggested that the higher birthweight seen for all these cohorts compared with the WHO standard may be explained by prior maternal undernutrition in some of the cohorts used to develop the WHO standard.^25^ However, a recent study suggested that birth weights across diverse countries, including some of those included in the WHO Multicentre Growth Reference Study (MGRS) cohorts, were not significantly different by country.^5^

Though born heavier, the infants here initially lost weight relative to the WHO standard and then regained it, causing a trough to appear in the Z score growth curve. This has been described in other cohorts,^5^ ^8^ and was influential in the UK rejecting the use of the WHO standard at birth.^26^ This pattern is the mirror image of the way breastfed infants used to grow on charts based mainly on bottle-fed infants and though seen in all datasets, its depth decreases over time.^2^ ^5^ We do not have information about breastfeeding rates in the routine cohorts but most will have been lower than the WHO sample.^27^ However, breastfeeding initiation rates have risen in the UK over the past 50 years from 36% in 1970 to over 70% in the early 21st century (UK)^28–30^ while when the Tampere cohort were born, 93% of Finnish infants were initially breastfed.^20^ The CIGS dataset, a more selective research sample, showed the shallowest trough and had higher rates of initial breastfeeding (75%) and breastfeeding to 6 months (48%).^31^ This pattern by era may also reflect changes in formula milk composition which has increasingly mirrored the nutritional content of breast milk.^32^

In contrast, the later rise relative to the standard did not show the same trend over time, suggesting that it may be unrelated to breastfeeding. While observational studies have found an association between use of breast milk substitutes and faster weight gain later in infancy^2^ ^31^ this effect was not seen in the Belarus trial of breastfeeding promotion.^33^ The sampling process used to construct the WHO standard could in principle mean that the MGRS cohorts were lighter than the general population. Children who were either outliers or not breastfed to 12 months were excluded from the sample^1^ and it has been shown that larger children are more likely to cease breastfeeding early.^33^ ^34^ However, the WHO MGRS group did not find any systematic difference in size between those included and excluded (personal communication De Onis, email communication, 2006).

Measurement error might also play a role, since the MGRS cohorts were measured using the same research protocols, where all clothes were removed or adjusted for.^4^ ^35^ The present cohorts mainly used routine data collected by health professionals; UK and Finnish guidelines recommend weighing infants naked up to the age of 2 years,^36^ but parents of older infants may be reluctant to comply, leading to higher weights at later ages. The CIGS cohort where all infants were weighed naked to a research protocol tracks closest to the WHO standard beyond 6 months. However, another study that compared routinely collected measurements with research measurements of the same children found little difference in weight.^37^

## Conclusions

The overall fit to the WHO weight standard of these cohorts ranged from excellent to adequate, so that use of the WHO standard is unlikely to introduce major bias in the assessment of individual children. Some of the more subtle variation in fit is likely to reflect variations in the levels of breast and formula feeding, as well as the composition of formula milk, in different eras. However, the lack of a consistent trend for weight gain after the initial weeks suggests that the higher average weight gain seen in North European cohorts cannot simply be explained by increasing rates of obesity in later childhood and adulthood.
